# Comparison and Optimization of Different Methods for the *In Vitro* Production of *Plasmodium falciparum* Gametocytes

**DOI:** 10.1155/2012/927148

**Published:** 2012-03-15

**Authors:** María Roncalés, Jaume Vidal-Mas, Didier Leroy, Esperanza Herreros

**Affiliations:** ^1^Malaria Discovery Performance Unit, GlaxoSmithKline R&D, Tres Cantos Medicine Development Campus, C/Severo Ochoa No. 2, Tres Cantos, 28760 Madrid, Spain; ^2^Medicines for Malaria Venture. 20, route de Prè-Bois. 1215 Geneva 15, Switzerland

## Abstract

The generation of sexually committed parasites (gametocytogenesis) is poorly understood in malaria. If the mechanisms regulating this process were elucidated, new opportunities for blocking malaria transmission could be revealed. Here we compare several methods described previously for the *in vitro* production of *Plasmodium falciparum* gametocytes. Our approach relies on the combination of several factors that we demonstrated as impacting on or being critical to gametocytogenesis. An improved method has been developed for the *in vitro* production of *P. falciparum* gametocytes as the first step toward obtaining adequate numbers of pure gametocytes for *in vitro* studies, such as, for example, the identification of transmission blocking drugs.

## 1. Introduction

Malaria parasites are capable of modulating the proportion of blood stage asexual parasites that develop into nonreplicating transmission stages (gametocytes). Unfavorable conditions for asexual replication, and thus future transmission opportunities, stimulate an increase in short-term gametocytogenesis [1–3]. Many reports suggest that there is a trigger for the production of gametocytes and various factors have been proposed as possible promoting factors [4, 5]. Carter and Miller demonstrated that environmental conditions directly modulate the rate of gametocyte production by *P. falciparum *in culture [6]. We echo the authors suggesting that multiple factors are likely to promote gametocytogenesis: higher parasitemia, grade of synchrony of the culture, hematocrit of the culture, quality and age of the blood, addition of lysed erythrocytes, elimination of asexual stages by different methods (sorbitol treatment or addition of pyrimethamine), media modification due to cell products and parasite metabolism, and so forth [4, 7–11]. These factors support the hypothesis that increased gametocyte production is a general response to retarded asexual growth in unfavorable and stressed conditions. Finally, it is crucial to keep a sufficient, yet fine balance, between inducing stressed conditions in asexual cultures (to maximize gametocytogenesis) and minimizing cell death [2, 12]. The aim of our study was to consider all these factors involved in gametocytogenesis to set up an optimized and efficient method whereby a reasonable number of young and mature *P. falciparum *gametocytes could be produced *in vitro*. Such a system is an absolute requirement for the development of *in vitro* assays measuring the gametocidal activity of new antimalarial drugs and would also constitute a starting point for the *in vitro* production of sporogonic stages.

## 2. Materials and Methods

### 2.1. Culture Medium

RPMI 1640 with 25 mM HEPES, sodium bicarbonate, and glutamine (GIBCO ref: 52400-025), supplemented with 10% of pooled human sera AB (14-490 E Cambrex) and 0.15 mM of hypoxanthine (from HT supplement x50, GIBCO ref. 41065). Human sera were decomplemented for 30 minutes at 56°C, aliquoted and stored frozen at −20°C until use. The complete medium was usually prepared fresh just before use and prewarmed to 37°C.

### 2.2. Red Blood Cells (RBCs)

Red blood cell stock suspensions were prepared from whole-blood bags from incomplete blood donations, provided by the Spanish Red Cross (less than 25 days after sampling). Whole blood was aliquoted and stored at 4°C.

To prepare red blood cells, whole blood was centrifuged and washed 3 times with RPMI without serum by centrifugation (10 minutes at 2000 rpm). The upper phase, containing white blood cells, was eliminated. Washed red blood cells were kept as a 50% suspension in complete medium and stored for a maximum of 4 days at 4°C.

### 2.3. Parasites

*P. falciparum* 3D7, Dd2, FCR3, HB3, W2, and NF54 strains were obtained from the MR4 Resource Center (ATCC) and maintained in complete medium at 5% of hematocrit value in continuous culture using a method adapted from Trager and Jensen [13]. Parasitemia was calculated by counting the percentage of parasitized erythrocytes using light microscopy. Gametocytemia was calculated by counting as a percentage the number of gametocytes (determining each specific stage) per number of erythrocytes (5000 RBCs). Thin blood films were made every day from each culture flask, fixed with methanol, and stained for 10 minutes in Giemsa (Merck, ref: 1.09204) at 5% in buffered water pH 7.2 (Buffer tablets, Merck). The culture was maintained in 75 cm^2^ culture flasks (canted neck, Corning) at 37°C, in a low oxygen atmosphere (5% CO_2_, 5% O_2_, 95% N_2_).

### 2.4. Synchronic Treatment

To establish synchrony, cultures were treated by the method described by Lambros and Vanderberg based on sorbitol treatment [14]. 

#### 2.4.1. Sorbitol Solution

 5% (w/v) of sorbitol (Sigma S 6021) in cell culture grade water (Sigma W3500) was used.

#### 2.4.2. Sorbitol Treatment to Synchronize Cultures

A method of synchronization based on the differential permeability of parasitized RBC membranes. Prewarm an aliquot of 5% D-sorbitol, complete medium and RPMI at 37°C. Remove most of the used medium from the flask and transfer the remaining culture to a 15 mL tube. Centrifuge for 5 minutes at 1800 rpm at room temperature and annotate the pellet volume. Transfer to a 50 mL tube and wash twice with prewarmed RPMI centrifuging for 10 minutes at 1800 rpm at room temperature. After the last wash, remove as much supernatant as possible without aspirating the cells. Resuspend in 5 volumes of pellet of prewarmed sorbitol solution. Homogenate with the pipette or by soft vortexing and incubate 5 minutes at 37°C. Centrifuge for 5 minutes at 1800 rpm. Remove supernatant and wash once with 25 mL of complete medium. Transfer pellet to a 15 mL centrifuge tube and wash with 10 mL of complete medium, remove supernatant, adjust pellet to 50% of hematocrit value, and use it to inoculate a culture in a 75 cm^2^ culture flask, adding fresh RBCs to maintain hematocrit value at 5% for synchronic cultures and at 2% for gametocyte production cultures.

#### 2.4.3. Sorbitol Treatment to Eliminate Asexual Stages

Use the same procedure of synchronization described above, except the sorbitol pulse (2.5 volumes of pellet) and the last step, in which the pellet is adjusted to 2% of hematocrit value without the addition of fresh RBC.

### 2.5. Thawing of Malaria Parasite Stabilates

Remove vials from liquid nitrogen and thaw immediately in a 37°C waterbath. Transfer stabilate to a 50 mL tube and estimate volume. Add 12% (w/v) NaCl in PBS (0.2 mL per mL of stabilate), dropwise very slowly, constantly swirling tube. Add slowly 1.6% (w/v) NaCl in PBS (10 mL per mL of stabilate). Add slowly 0.9% (w/v) NaCl with 0.2% (w/v) glucose in PBS (10 mL per mL of stabilate). Centrifuge the resulting solution for 5 minutes at 1800 rpm. Remove supernatant and resuspend the pellet in 10 mL incomplete RPMI, adding it slowly at first. Centrifuge for 5 minutes at 1800 rpm and remove supernatant. Resuspend cells in 6 mL complete RPMI culture medium, transfer to a 25 cm^2^ culture flask and incubate at 37°C [15].

### 2.6. Akompong Protocol

Asexual parasite cultures were diluted to a 0.2% parasitemia and 6% hematocrit value with fresh RBC in a 75 cm^2^ culture flask (day 0). On day 3, the cultures were diluted from a 6% to a 3% hematocrit with culture medium and then maintained for the next 18 days with daily medium changes. No erythrocytes were added to the cultures after day 0. Giemsa-stained slides of the cultures were prepared daily to monitor parasitemia and gametocytemia [9].

### 2.7. Bennett Protocol

Asexual parasite cultures were diluted to a 1% parasitemia and 2% hematocrit value with hemoglobin-depleted RBCs in a 75 cm^2^ culture flask with 20 mL of culture medium. The media were changed daily over 8 days. [Hemoglobin depleted: Red blood cells were incubated for 30 minutes with hypotonic solution (5 mM HEPES, 11 mM glucose, 2 mM ATP in bidistilled water) to reduce their hemoglobin content]. Giemsa-stained slides of the cultures were prepared daily to monitor parasitemia and gametocytemia [11].

### 2.8. Chavalitshewinkoon-Petmitr Protocol

Cultures treated with two serial sorbitol treatments were subcultured at 0.5% or 1% of the initial parasitemia (depending on the strain used) and 2% of hematocrit value in a 75 cm^2^ culture flask with 20 mL of culture medium. The culture medium was changed on days 4 and 6 and daily from days 8 to 15. On days 9, 10, and 11, 2.5 pellet volumes of 5% (w/v) sorbitol solution were added for 5 minutes once a day to remove asexual remaining forms and select only gametocytes. Giemsa-stained slides of the cultures were prepared daily to monitor parasitemia and gametocytemia [7].

### 2.9. Chavalitshewinkoon-Petmitr + Bennett Protocol

Combination of both protocols using the culture settings of *Chavalitshewinkoon-Petmitr *procedure complemented with the hemoglobin depleted RBCs from *Bennett* protocol [7, 11].

### 2.10. Schneweis Protocol

Asexual parasite cultures were synchronized and diluted to a 0.5–1% initial parasitemia and 10% hematocrit value in a 75 cm^2^ culture flask with 20 mL of culture medium. The medium was changed daily. After 48 hours of culture, lysed uninfected erythrocytes (LUE) suspension was added at final concentration of 20% to induce gametocytogenesis. Giemsa-stained slides of the cultures were prepared daily to monitor parasitemia and gametocytemia [4].

### 2.11. Lysed Uninfected Erythrocytes (LUE) Suspension

For lysis of erythrocytes, washed and pelleted human RBCs were frozen at −10°C for 20 minutes and warmed up again at 37°C for 20 minutes. This was repeated twice. To reduce toxicity, we diluted one-part lysed erythrocytes with one-part RPMI medium. This LUE suspension was ready to be added to the cultures.

### 2.12. Chutmongkonkul Protocol

Asexual parasite cultures were synchronized and diluted to a 0.75% initial parasitemia and 2% hematocrit value in a 75 cm^2^ culture flask with 20 mL of culture medium. The medium was changed on day 4 of production. On day 5, 40 *μ*L of pyrimethamine (24.87 ng/mL) was added to eliminate asexual stages. On day 7, the culture was washed twice with 40 mL of RPMI (1800 rpm, 5 minutes) in order to reduce the drug amount. The medium was changed daily from days 6 to 15. Giemsa-stained slides of the cultures were prepared daily to monitor parasitemia and gametocytemia [8].

### 2.13. Chutmongkonkul + Schneweis Protocol

Combination of both protocols using the culture settings of *Chutmongkonkul *procedure complemented with the addition of LUE suspension at 20% on day 4, from *Schneweis *protocol [4, 8].

### 2.14. Chavalitshewinkoon-Petmitr + Schneweis Protocol

Combination of both protocols using the culture settings of *Chavalitshewinkoon-Petmitr *procedure complemented with the addition of LUE suspension at 20% on day 4, from *Schneweis *protocol [4, 7].

## 3. Results and Discussion

Manipulation of the culture conditions and addition of pharmacological agents could increase gametocyte numbers or apparently initiate gametocytogenesis [3]. For example, the addition of red cell lysate [4, 6], presence of human serum [16], or some antimalarial drugs such as chloroquine [1] could increase gametocyte production. Young et al. proposed that synchronized sexual development was induced by a sudden increase to the hematocrit of a fast growing ring stage culture [17]. Carter et al. affirmed that it is necessary for the asexual blood stage to reach high densities in the culture and become stressed to convert to the production of large numbers of gametocytes [18]. Ifediba and Vanderberg concluded that gametocyte production could be enhanced by reducing the hematocrit for part of the culture period [10]. Buchholz et al. affirmed that sexual conversion could be induced *in vitro* by a sudden decrease in hematocrit concentration and a high parasite load, reflecting conditions of physiological stress in the human host that are known to correlate with increased numbers of transmission stages in blood circulation [19]. It is possible that there may not be a single mechanism and that multiple factors may contribute to the decision to form gametocytes [3].

Considering some of these aspects, existing static culturing protocols that stimulate gametocytogenesis typically involve seeding flasks with a low parasitemia (0.2%) and high hematocrit value (6%) on day 0 of gametocyte production. On day 3, the cultures were diluted from a 6% to 3% hematocrit (increasing the medium volume to stimulate healthy growth of the induced gametocytes) and the medium in the flasks was then changed daily over the next 18–20 days without any addition of fresh uninfected erythrocytes, as gametocyte production is facilitated under these conditions [9, 10, 12]. Moreover, Schneweis et al. and Bennett et al. proposed, respectively, that adding lysed erythrocytes (at a final concentration of 20%) to the culture medium or reducing the hemoglobin content of the erythrocytes using a treatment with hypotonic solution for 30 minutes would enhance *in vitro *gametocyte production [4, 11]. Bennett et al. also suggests that not all laboratory strains produce gametocytes with the same occurrence even if these strains are stimulated by the same induction method [11]. Another factor to consider is the correlation between asexual parasitemia and commitment to sexual development. Carter and Miller concluded that environmental conditions directly modulate the rate of gametocyte production by *P. falciparum *in culture [6]. However, the actual factors responsible and the way in which they can modify the level of sexual conversion are still to be determined [2, 3]. It is commonly accepted that, when the level of asexual parasitemia decreases, the rate of parasite conversion to gametocytes increases [2, 5]. Related to this, Chutmongkonkul et al. described the use of pyrimethamine at low concentrations, 40 *μ*L of pyrimethamine (24.87 ng/mL), to eliminate asexual stages and Chavalitshewinkoon-Petmitr et al. proposed treatment with sorbitol (at 5% w/v on days 9, 10, and 11 of gametocyte production) for the inhibition and elimination of asexual parasites [7, 8]. Considering all the factors that could modulate gametocytogenesis, we have compared and combined some of the protocols mentioned previously using different *P. falciparum *strains to establish the best *in vitro *approach for an efficient production of *P. falciparum* gametocytes from stages I to V. Various conditions used in each experiment and their results are summarized in Table 1.

First, we compared the reduction of hematocrit, the influence of hemoglobin content and the decrease of asexual parasites by sorbitol treatment as enhancers of gametocytogenesis, using synchronous cultures of *Plasmodium falciparum* strains 3D7 and Dd2 (experiment 1) [7, 9, 11]. The results we obtained showed that reducing the number of asexual parasites in culture was the most efficient strategy to produce higher numbers of pure gametocytes. In contrast, reduction of hematocrit did not produce the gametocytemia that had been described by previous authors [9, 10, 17]. The influence of hemoglobin content was associated with hypergametocytemia within 2 to 4 days of gametocyte production. Although we cultured parasites with more hemoglobin depletion than *Bennett* (40% of hemoglobin), we were unable to reach hypergametocytemia in either the 3D7 or Dd2 strain and could not completely eliminate asexual parasites from the culture. Alternatively, we combined the decrease of asexual parasites with the reduction of hemoglobin content and analyzed the outcome of both approaches when applied alone or in combination. The initial parasitemia of the gametocyte production culture was also investigated (experiment 2). Although the best results, considering the percentage of parasitemia (1.64% ± 0.24), were obtained using the combined strategy, this approach was discarded as it produced a mix of asexual parasites and gametocytes. In contrast, with the *Chavalitshewinkoon-Petmitr *protocol only gametocytes were observed, due to the elimination of asexual parasites by sorbitol treatment [7, 11]. The initial parasitemia of the gametocyte production culture must be established taking into account the synchrony and growth ratio of each strain in order to keep the balance between inducing stressed conditions and minimizing cell death. In previous studies (data not shown), we realized that synchronic cultures with the highest initial parasitemia could compromise the reinvasion process and survival of the parasites. As Petmitr et al. suggest, low initial parasitemia increased the numbers of gametocytes meanwhile higher parasitemia produced fewer gametocytes [20]. Considering the grade of synchrony and the growth ratio of the 3D7 and Dd2 strains, we concluded that the best initial parasitemia on gametocyte production was 0.5% for these specific strains. The production of gametocytes from different synchronized strains (3D7, Dd2, FCR3, HB3, W2, and NF54) using the *Chavalitshewinkoon-Petmitr *method (with the modification of the initial parasitemia) was also studied (experiment 3) [7]. The highest gametocytemia was obtained with the NF54 strain (0.75%  ± 0.20). As suggested by Bennett et al., gametocyte production was clearly strain dependent [11]. Several authors affirm that recently isolated strains are more likely to form gametocytes than strains that have been in culture for long periods of time [21, 22]. Mons and Van Der Kaay affirmed that gametocyte generation of *P. berghei* increases after the cryopreservation of the cultures [23]. Ponnudurai et al. concluded that the potential for producing NF54 gametocytes is undiminished during storage of stabilates in the cryopreserved state [21]. To test this statement, we launched a new production of gametocytes according to the *Chavalitshewinkoon-Petmitr *protocol [7] and using NF54 and W2 strains just released from cryopreservation (experiment 4) [15]. As shown in Table 1, gametocyte numbers were higher when prepared from just thawed NF54 parasites. We observed that NF54 is a strain that retains the capacity to produce gametocytes after being cryopreserved. Although there were no differences between synchronic and just thawed strains (*P* value 0.06 for W2 and 0.37 for NF54), we preferred to use storage stabilates of NF54 to avoid the reduction of gametocyte production due to passage numbers. Taking into account all the previous results, the reduction of asexual parasites was considered a key factor in promoting gametocytogenesis. To study this factor, we compared two protocols based on treatment by either sorbitol (*Chavalitshewinkoon-Petmitr *protocol) or pyrimethamine (*Chutmongkonkul *protocol) for the reduction of asexual parasites of the culture (experiment 5) [5, 6]. The original protocols of *Chavalitshewinkoon-Petmitr *and *Chutmongkonkul *were run in parallel using the NF54 strain [7, 8] and combining both treatments with the addition of lysed uninfected erythrocytes (amplifying the accumulation of parasite metabolism and cell products) as another factor that could influence the sexual development of the parasite (*Schneweis *protocol) [4]. As described in Table 1, the highest gametocytemia was obtained with the new combination of protocols (experiment 5), which considered the decrease of asexual stages by sorbitol treatment and the addition of lysed uninfected erythrocytes as the main factors on gametocyte production. On the other hand, the *Chutmongkonkul *protocol (which reduced asexual parasites through the addition of pyrimethamine) had less effect (on gametocyte production), even when it was supplemented by the addition of LUE. With the new combined method, we reached a gametocytemia of 1.02%  ±  0.05 where asexual parasites were completely eliminated. This successful approach was reproduced several times to assess its reliability using the NF54 strain where conditions of initial parasitemia (0.75%) and hematocrit (2%) were also adjusted (experiment 6). Average production ranged from 0.93% to 1.18% of gametocytemia. We observed significant differences (*P* value 0.0034) between this new method and basal production of gametocytes on a control experiment (gametocyte production with no addition of LUE or pulse sorbitol with the NF54 strain). This optimized protocol combined two key factors shown to enhance gametocytogenesis: the addition of lysed uninfected erythrocytes (LUE) and the complete elimination of asexual stages through sorbitol treatment. We decided not to use the elimination of asexual stages through pyrimethamine treatment for two reasons: (i) it does not completely eliminate the asexual stages and (ii) as Chutmongkonkul et al. described, it kills the young gametocyte stages [8]. As Bennett et al. suggested, we observed that not all laboratory strains produce gametocytes to a similar extent even if they are stimulated by the same induction method [11]. The level of culture synchrony was increased by thawing the parasites and was an additional factor we integrated to improve the production of a healthy population of gametocytes with maturation capability, avoiding the reduction of gametocyte production due to passage number of the cultures.

In summary, we compared previously described protocols and set up an optimized method for an improved *in vitro* production of *P. falciparum *gametocytes, which consisted of two main steps: thawing an aliquot of cryopreserved *P. falciparum *NF54 parasites (that retain their capability of producing gametocytes) and synchronizing the resulting culture by two serial sorbitol treatments (48 hours each) in order to start gametocyte production with the highest amount of ring stages on the culture. A certain percentage of the ring forms that appear in culture are committed to developing into gametocytes; this percentage (conversion rate) was calculated using the following formula: [number of stage II gametocytes that appear in the culture 2 days after the ring forms (per 5000 RBCs)/number of ring forms (per 5000 RBCs)]*100; the conversion rate from rings to gametocytes was between 11% and 23% [24]. At the end of the second sorbitol treatment, the culture was adjusted to 0.75% of the initial parasitemia and a 2% hematocrit value (day 1 of production). On day 4, a suspension of lysed uninfected erythrocytes (LUE) was added at a final concentration of 20% to induce gametocytogenesis. The culture medium was changed on days 4 and 6 and daily from days 8 to 15 to remove cell debris. Sorbitol treatments (2.5 pellet volumes of sorbitol solution) on days 9, 10, and 11 were carried out to remove remaining asexual forms and to enrich the cultures in gametocytes. Neither the stage nor the development of gametocytes was affected by the sorbitol treatments. *P. falciparum* NF54 was clearly identified as the strain that produced the highest gametocytemia (1.18 ± 0.07) with this protocol, producing the complete development of each gametocyte stage (I–V) and excellent reproducibility.

**Figure 1:**
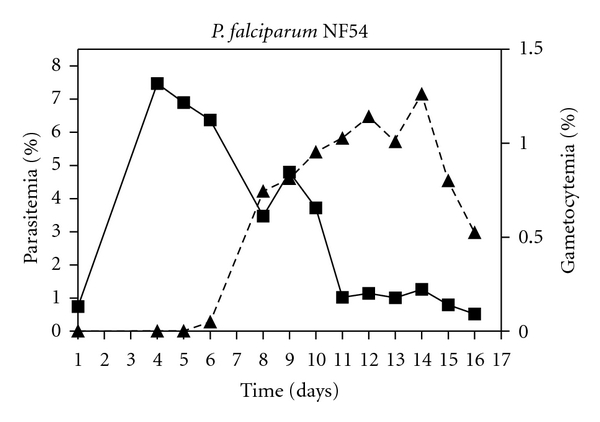
Time course of *in vitro *production of *P. falciparum *NF54 gametocytes. Percentage of total parasitemia (■) and percentage of gametocytes (▲) are represented.

The time course of *P. falciparum *NF54 gametocytes produced *in vitro *following the protocol described above is shown in Figure 1.

**Figure 2:**
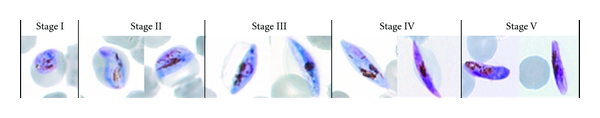
*In vitro P. falciparum *gametocytes obtained with this optimized protocol. Gametocytes stages (maturity) based on the Carter description: stage I, stage II, stage III, stage IV, and stage V.

Total parasitemia increased by 7-8% up to day 4 when, due to stress conditions, gametocyte production started to raise. We observed that as asexual stages decreased on day 8, the percentage of gametocytes increased until day 14, reaching a gametocytemia of 1–1.2%. *In vitro *gametocyte production required approximately 15 days. During this time, it was possible to define the specific day each gametocyte stage appeared and their maturation from early (young gametocyte) to late (mature gametocyte) stages. On day 6 and day 11 of production, we obtained stage II and stage III, respectively. The gametocytes then matured up to stage IV on day 14 and stage V on day 16 (Figure 2). We classified the gametocyte stages (I–V) according to the description made by Carter and Miller [6].

Considering key factors involved in gametocytogenesis, such as the reduction of asexual stages and the addition of lysed uninfected erythrocytes, here we propose an optimized and efficient strategy for the *in vitro* production of *P. falciparum *gametocytes based on the comparison of different protocols. This new method will allow the accurate determination of the gametocidal activity of new antimalarial drugs against each developmental phase of the sexual blood stage (study in progress, data not shown) and will also be the starting point for the production of the subsequent sporogonic stages.

## Figures and Tables

**Table 1:** Factors involved in gametocytogenesis studied and corresponding gametocyte production.

Name	Conditions studied	Protocol	% Parasitemia	% Hematocrit	Strain	% Gametocytemia
Experiment 1	Reduction of hematocrit	[1]	0.2	6 to 3	3D7-Syn.	0.08 ± 0.03
					Dd2-Syn.	0.19 ± 0.02
	Hemoglobin content	[2]	1	2	3D7-Syn.	0.25 ± 0.09*
					Dd2-Syn.	0.22 ± 0.14*
	Decreased asexual parasites	[3]	1	2	**3D7-Syn.**	0.37 ± 0.02
					Dd2-Syn.	0.25 ± 0.30

Experiment 2	Decreased asexual parasites	[3]	1	2	3D7-Syn.	0.42 ± 0.05
			0.5	2	**Dd2-Syn.**	0.51 ± 0.61
	Decreased asexual parasites	[3, 2]	1	2	3D7-Syn.	0.60 ± 0.26*
	Hemoglobin content		0.5	2	Dd2-Syn.	1.64 ± 0.24*

Experiment 3	Decreased asexual parasites Different strains	[3]	0.5	2	3D7-Syn.	0.34 ± 0.07
					Dd2-Syn.	0.49 ± 0.07
					FCR3-Syn.	0.00 ± 0.00
					HB3-Syn.	0.04 ± 0.01
					W2-Syn.	0.29 ± 0.05
					**NF54-Syn.**	0.75 ± 0.20

Experiment 4	Decreased asexual parasites Just thawed strains	[3]	1	2	**NF54 thawed**	0.90 ± 0.09
					W2 thawed	0.67 ± 0.24

Experiment 5	Decreased asexual parasites	[3]	0.75	2	NF54 thawed	0.62 ± 0.10
	Decreased asexual parasites + LUE	[3, 4]			**NF54 thawed**	1.02 ± 0.05
	Decreased asexual parasites	[5]			NF54 thawed	0.44 ± 0.06*
	Decreased asexual parasites + LUE	[5, 4]			NF54 thawed	0.48 ± 0.19*

Experiment 6	Decreased asexual parasites + LUE	**New Optimized** **Protocol**	0.75	2	**NF54 thawed**	1.18 ± 0.07

Footnotes and abbreviations: The percentage of gametocytemia was expressed as a mean and standard deviation of two replicates of three independent experiments (*P* values of unpaired *T*-test were calculated using GraphPad Prism). Best production is highlighted in bold. Syn-Synchronized cultures; Thawed-just thawed cultures; *Presence of asexual parasites and gametocytes in the culture. Legend in the protocol column corresponds to protocols [1] (see [9]), [2] (see [11]), [3] (see [7]), [4] (see [4]), and [5] (see [8]).
